# Central retinal vein occlusion complicating spontaneous dural carotid-cavernous fistula after phacoemulsification

**DOI:** 10.3205/oc000117

**Published:** 2019-07-16

**Authors:** Mahmoud Mohamed Farouk, Engy Mohamed Mostafa, Amr Mounir

**Affiliations:** 1 Sohag Faculty of Medicine, Ophthalmology Department, Sohag, Egypt

**Keywords:** dural carotid-cavernous fistula, central retinal vein occlusion, phacoemulsification

## Abstract

In this report, we are presenting a case of a 49-year-old female complaining of defective vision in the left eye. The main complaints were: pain, redness, mild proptosis, and high intraocular pressure. She had a history of uneventful phacoemulsification surgery 3 months prior to presenting to us. Investigations revealed a macular edema caused by central retinal vein occlusion and computed tomography angiography showed an early opacified left cavernous sinus with a dilated superior ophthalmic vein along with a fistula between the meningeal branches of the carotid arteries and the cavernous sinus. Improvement of ocular symptoms was achieved after endovascular treatment by transarterial and transvenous embolization.

## Introduction

Carotid-cavernous fistula (CCF) is an abnormal communication between a previously normal carotid artery and the cavernous sinus [1]. A spontaneous rupture of an existing aneurysm or atherosclerotic artery is an uncommon cause of CCF which is called indirect or dural CCF which usually occurs in postmenopausal, hypertensive females [2]. Indirect fistulas are usually manifested by mild symptoms, with insidious onset, mild orbital congestion, and mild degree proptosis with low or no bruit [3]. We report a case of spontaneous dural CCF presented by central retinal vein occlusion (CRVO) with macular edema after uneventful phacoemulsification surgery.

## Case description

A 49-year-old female presented to the outpatient clinic of the ophthalmology department of Sohag University, Egypt with gradual defective vision in the left eye, mild ocular pain and redness which started 2 months ago. The patient had an uneventful phacoemulsification in the same eye 3 months ago. She was known to be diabetic and hypertensive on regular medical therapy.

Initially, the patient was diagnosed by her physician as having postoperative uveitis with secondary glaucoma and was managed by topical steroids and anti-glaucoma medications without any improvement.

**Figure 1:**
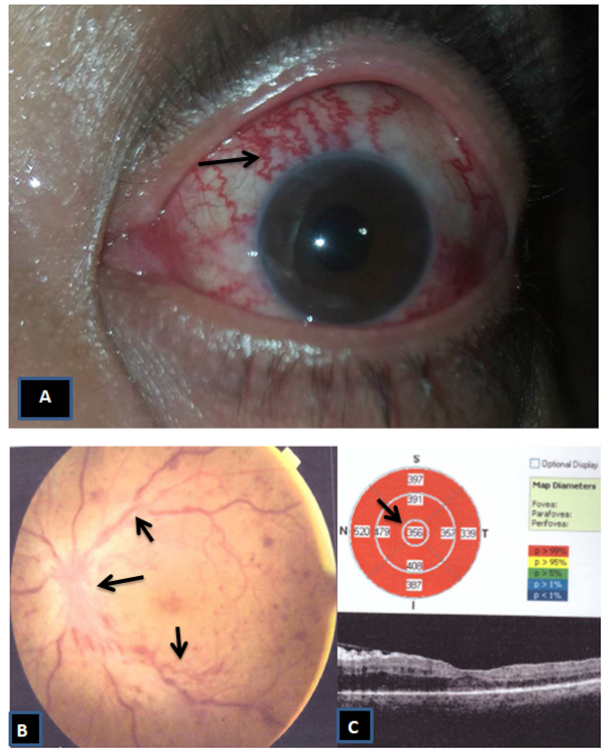
A) Conjunctival injection with corkscrew vessels and mild lid edema in the left eye. B) Picture of central retinal vein occlusion with dilatation of the retinal veins, scattered dot retinal hemorrhages and optic disc margin blurring in the left eye. C) Macular edema in the affected eye with central macular thickness (CMT) 356 µm.

We re-evaluated her with full ophthalmological examination, the best corrected visual acuity (BCVA) was 0.5 (in decimal notion) in the right eye and 0.2 in the left eye. Extraocular movements were full and free in both eyes. There was a mild periorbital edema and redness around the left eye (Figure 1 (Fig. 1)). Pupils of both eyes were equal in size and reactive to light without relative afferent pupillary defect. With Hertel’s exophthalmometry measurement, proptosis wasn’t detected in the right eye (17 mm) while there was mild proptosis in the left eye (23 mm).

Slit lamp examination showed a cortical cataract (LOCS: C2) in the right eye which explains the decreased visual acuity. While the left eye showed a significant conjunctival injection with episcleral vessel dilatation in the form of a corkscrew (Figure 1A (Fig. 1)). The implanted lens in the left eye was in place with a clear anterior chamber. Intraocular pressure was 16 mmHg in the right eye and 27 mmHg in the left eye by Goldman applanation tonometry. No bruit was heard mostly because it was a low flow CCF. Extraocular muscles were also spared mostly for the same reason.

Fundus examination showed a picture of central retinal vein occlusion with dilatation of the retinal veins, scattered retinal hemorrhages, and an edematous optic disc with blurring of its margin in the left eye and with normal fundus in the right eye (Figure 1B (Fig. 1)). Optical coherence tomography (OCT) showed a macular edema in the affected eye with a central macular thickness (CMT) of 356 µm (Figure 1C (Fig. 1)).

Based on clinical signs of the dilated episcleral vessel, proptosis, periorbital swelling in the left eye, and being refractory to steroid therapy by the primary physician, a carotid-cavernous fistula was suspected and further investigations were needed to confirm the diagnosis.

**Figure 2:**
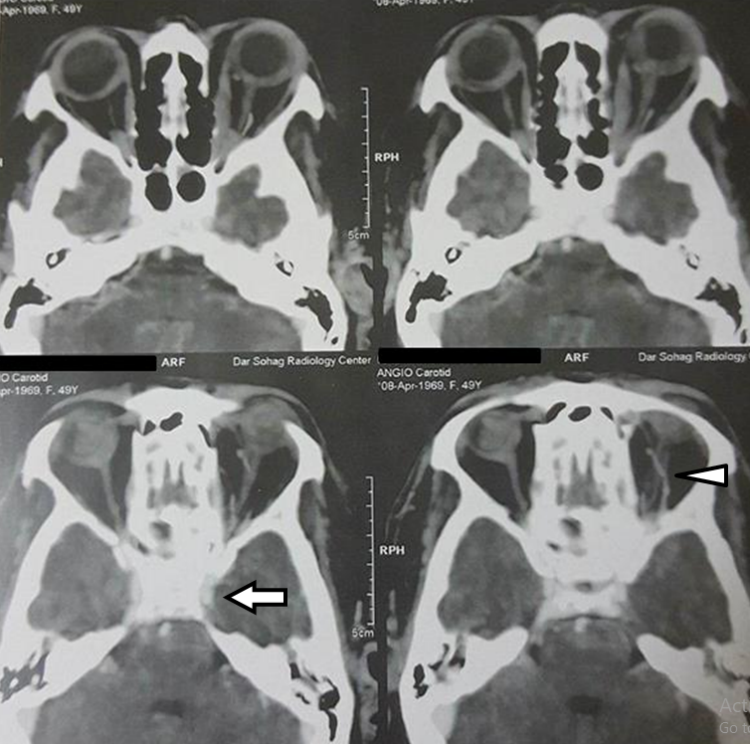
Minute small vessels around the early opacified left cavernous sinus (arrow) with dilated superior ophthalmic vein (arrow head)

Computed tomography angiography (CT-A) was done (Figure 2 (Fig. 2)) with positive findings. Images showed minute small vessels (fistulas) around the early opacified left cavernous sinus with a dilated superior ophthalmic vein.

On confirming the diagnosis of dural CCF, the patient was referred to the neurosurgery department where endovascular treatment by transarterial and transvenous embolization was done.

After treatment, an improvement of the ocular symptoms occurred within one week with relief of conjunctival injection and lid swelling. Dorzolamide/Timolol combination eyedrop was prescribed twice daily until the IOP dropped to 16 mmHg. In addition, the patient received 3 intravitreal injections of Bevacizumab (one injection monthly) for management of the macular edema. The macular edema improved to a CMT of 192 µm one month after cessation of injections and BCVA of 0.7.

## Discussion

Carotid-cavernous fistula (CCF) is classified anatomically into two types; either direct or indirect (with high-flow or low-flow fistulas) due to either traumatic or spontaneous causes [4], [5]. CCF are misdiagnosed in many conditions especially the dural type leading to significant visual problems and further increasing patient’s morbidity [6].

The dural CCF has a relatively benign clinical course compared with the direct type [7]. Recovery of the dural type resulting from spontaneous obstruction of shunts occurs in about 50% of cases [8].

In our report, we present a case of misdiagnosed dural CCF which has been complicated by central retinal vein occlusion presenting after a short duration of phacoemulsification surgery. The subtle signs in this case could be explained by being a low flow dural CCF.

Central retinal vein occlusion complicating spontaneous dural CCF has been reported in few reports, but it is not a common manifestation of CCF [9], [10], [11].

In a study by Nagaki et al. [12], a case of carotid-cavernous fistula associated with choroidal detachment after cataract surgery was reported. Dural CCF after cataract surgery is a rare condition which can be explained by the surgical trauma effect which leads to a spontaneous rupture of small meningeal arteries supplying the dural wall of the cavernous sinus while the internal carotid artery itself may remain intact [13].

The dural CCF may have existed before surgery, yet the lack of signs prior to surgery makes it a remote possibility. The proposed explanation for the development of CCF after the cataract surgery is retrobulbar anaesthesia given prior to the surgery which may have caused high venous pressure resulting in venous stasis of the superior ophthalmic vein and hence triggering dural CCF. This would have led to retrograde thrombosis towards the central retinal vein as well. Retrobulbar anaesthesia has been reported to cause central retinal vessel occlusion; artery occlusion much commoner than vein occlusion [14]. Whether the CCF caused CRVO or the other way round is difficult to confirm. Komiyama et al. [15] reported a case of CRVO favoring an explanation that sinus thrombosis may extend to the superior ophthalmic vein, producing CRVO. 

Carotid Doppler should have been ordered to detect any emboli for preventing any other underlying cause. Also orbital sonography would have been valuable to evaluate the superior ophthalmic vein.

To the best of our knowledge, this is considered the second reported case of CCF after intraocular surgery.

## Conclusions

In conclusion, spontaneous dural carotid-cavernous fistula can rarely occur as a complication of intraocular cataract surgery which can be presented by central retinal vein occlusion. Dural CCF could easily be misdiagnosed in many conditions leading to significant visual morbidity.

## Notes

### Competing interests

The authors declare that they have no competing interests.
